# Efficacy and Safety of a Dual‐Wavelength 589/1319 nm Laser for the Treatment of Acne Erythema: A Split‐Face Randomized Controlled Trial

**DOI:** 10.1111/jocd.70894

**Published:** 2026-05-05

**Authors:** Suphagan Boonpethkaew, Pimsiri Anansiripun, Warittha Maitrisathit, Yanisa Ratanapokasatit, Sonphet Chirasuthat, Panrudee Wechsuruk, Penpun Wattanakrai

**Affiliations:** ^1^ Division of Dermatology, Department of Medicine Ramathibodi Hospital, Mahidol University Bangkok Thailand

**Keywords:** 589/1319 nm solid‐state dual‐wavelength laser, acne erythema, acne treatment, acne vulgaris, *Aloe vera* gel, non‐ablative laser

## Abstract

**Background:**

The 589/1319 nm solid‐state dual‐wavelength (SSDW) laser, which targets cutaneous vasculature, may be an effective treatment for acne erythema (AE).

**Objectives:**

To compare the efficacy and safety of the 589/1319 nm SSDW laser with topical soothing *Aloe vera* (AV) gel for the treatment of AE.

**Patients and Methods:**

Thirty patients with bilateral AE were enrolled. One facial side received 6 sessions of 589/1319 nm SSDW laser treatment, while the contralateral side was treated with twice‐daily AV gel for 18 weeks. Patients were followed for 8 weeks after the final laser session. AE severity, acne severity, and adverse events were assessed.

**Results:**

Twenty‐nine patients completed the study. Both treatment modalities significantly reduced AE. The laser‐treated side demonstrated a faster clinical response, with a significant reduction compared to baseline at 2 weeks, whereas AV gel required 4 weeks to achieve a comparable effect (within‐group *p* < 0.05; no between‐group difference). At the 8‐week follow‐up, the response rate was 72% for the laser‐treated sides and 69% for the AV‐treated sides. AE improvement correlated with reductions in acne severity in the laser‐treated sides (*r*_s_ = 0.47, *p* = 0.03). Patients reported higher satisfaction with laser treatment up to 4 weeks after the final laser session. Average pain score for the laser treatment was 1.52 out of 10. No serious adverse events were observed.

**Conclusions:**

The 589/1319 nm SSDW laser may be an effective early adjunctive treatment for acne erythema, offering minimal discomfort with no downtime and may additionally improve acne severity.

## 1 Introduction

Acne erythema (AE) is a common concern both during active acne inflammation and after its resolution. It is characterized by erythematous macules or patches, often accompanied by telangiectasia [1]. While AE lesions may gradually improve over time, some persist even after inflammatory acne has resolved. AE is often considered cosmetically undesirable and can lead to significant patient frustration and psychological distress [2].

Various approaches, including topical and interventional therapies, have been used to treat AE [3]. Effective topical treatments include monotherapy and combination therapy with azelaic acid [4], tranexamic acid [5, 6], vitamin C gel [6], oxymetazoline [7, 8], and brimonidine tartrate solution [8], all of which show promising results in reducing AE. Aloe vera gel (AV gel), with its anti‐inflammatory, antioxidative, antibacterial (effective against *Cutibacterium acne*), anti‐pigmentation, and soothing properties, presents a promising topical treatment for AE [9, 10, 11, 12, 13]. Lasers and light‐based therapies have been frequently applied to treat AE; offering a quicker response and reduced acne sequelae [14, 15]. Commonly used devices include 585–595 nm pulsed dye laser (PDL), intense pulsed light (IPL), Q‐switched neodymium‐doped yttrium aluminum garnet (QS Nd: YAG), and diode lasers. These devices are effective in targeting dilated blood vessels and reducing redness [3]. Although PDL is the most widely used laser for treating AE, it still has minor side effects, such as pain, edema, erythema, purpura and post‐inflammatory hyperpigmentation (PIH) [16, 17].

The 589/1319 nm solid‐state dual‐wavelength (SSDW) laser is maintenance‐free and offers a minimally invasive treatment combining a PDL wavelength with a near‐infrared (NIR) wavelength. The 589 nm wavelength theoretically targets hemoglobin to reduce inflammation, while the 1319 nm wavelength targets water, generating heat to suppress sebaceous gland activity and promote tissue remodeling. Our recently published data has shown that the 589/1319 nm SSDW laser was effective in reducing inflammatory acne lesions which correlated with improvements in post‐inflammatory hyperpigmentation and decreased skin depression volume [18]. However, AE was not specifically focused.

In this study, we aimed to investigate the efficacy of the 589/1319 nm SSDW laser compared to a topical soothing agent for AE treatment.

## 2 Materials and Method

### 2.1 Research Design

This split‐face, single‐blinded, randomized controlled trial evaluated the efficacy of the 589/1319‐nm SSDW laser compared to topical soothing AV gel in treating AE. The study was conducted at Ramathibodi Hospital, Mahidol University, Bangkok, Thailand. It adhered to the Declaration of Helsinki and received ethical approval from the Human Research Ethics Committee of the Faculty of Medicine Ramathibodi Hospital, Mahidol University (COA.MURA2022/747). The trial was registered at www.thaiclinical trials.org as TCTR20230217005.

### 2.2 Sample Estimation and Subject Recruitment

Sample size was calculated for a paired (within‐subject) design. Based on an open‐access clinical study of laser treatment for post‐acne erythema reporting a Clinician Erythema Assessment Scale (CEAS) change (mean difference 0.79; paired *t* = 4.821; *n* = 22) [19], the standard deviation (SD) of within‐subject change was derived as $s_{d}=\overset{\prime }{d}\sqrt{n}/t\approx 0.77$. Assuming a two‐sided α = 0.05 (*Z* = 1.96) and 80% power (*Z* = 0.84), detecting a clinically meaningful difference of 0.42 CEAS points requires 26 participants $\left[n={\left(\left(1.96+0.84\right)\times 0.77/0.42\right)}^{2}\right]$. Allowing ~15% dropout, we planned to recruit 30 participants.

Inclusion criteria for this study comprised healthy male and female participants aged 18 to 50 years with Fitzpatrick skin types III to V with bilateral AE. Exclusion criteria included individuals with severe active acne vulgaris; pregnant or breastfeeding women; those who had taken oral and topical vitamin A derivatives within the past 3 months; individuals who had undergone any form of facial laser treatment within the past month; and those with a history of photosensitivity disorders, impaired wound healing, active skin infections, or skin cancer. and participants with known light sensitivity. All patients provided written informed consent, including consent for clinical photography before being enrolled.

### 2.3 Treatment Protocol

Block randomization was utilized to assign one facial side to receive 589/1319‐nm SSDW laser treatment, while the other side received topical AV‐gel. Patients received 6 sessions of 589/1319 nm SSDW laser (ADVATx, Advalight, Ballerup, Denmark) at 2‐weeks intervals on the randomized facial side, with follow‐up visits scheduled at 2, 4, and 8 weeks after the last laser session. AV gel (87.4%) (Radiara, Bangkok Lab and Cosmetic Public Company Limited, Bangkok, Thailand) was applied twice daily on the other facial side during the 18‐week study period. During the study period, patients were instructed to avoid sun exposure, heat, and friction and to apply sunscreen with SPF 50+. Patients were allowed to continue their topical acne medications, such as benzoyl peroxide, topical clindamycin, topical salicylic acid, topical vitamin A, excluding oral antibiotics, oral vitamin A derivatives, and azelaic acid. Adverse events were monitored throughout the study period. Photographs were taken on every visit.

Laser parameters were adjusted according to each subject's Fitzpatrick skin type as follows. For the initial 589 nm wavelength, skin types III were treated with a fluence of 40 J/cm^2^ to deliver 1400 J, while skin types IV–V received 20 J/cm^2^ to deliver 700 J, with transient erythema as the endpoint. This was followed by the 1319 nm wavelength treatment in which skin types I–III were treated at 40 J/cm^2^ to deliver 1200 J, and types IV–V treated at 20 J/cm^2^ to deliver 700 J, with a warm heat sensation as the endpoint. Laser pulses were applied using a 10 × 10 mm square scanner with a 0.25‐s repetition rate and a high‐density filling factor utilizing a gliding technique. No topical anesthesia was applied. A cooling gel was applied during the 589‐nm laser treatment.

### 2.4 Outcome Assessments

#### 2.4.1 Subjective Assessments

Improvement in facial AE and acne severity was assessed by two blinded dermatologists evaluating Visia camera photographs at baseline (week 0) and at weeks 2, 4, 6, 10, 12, 14, and 18. The erythema index was assessed on both sides of the face with the Clinician Erythema Assessment Scale (CEAS) (Table 1). The modified Investigator's Global Assessment (IGA) scale [18] was used to evaluate acne severity (Table 2). Patients completed questionnaires to evaluate their satisfaction of both treatment sides on weeks 10, 12, 14, and with a visual analog scale (VAS) ranging from 0 to 10 for self‐assessment of overall AE improvement (0 = worse, 5 = moderate satisfaction, and 10 = very satisfied). Pain related to laser treatment was also assessed by patients with VAS (0 = no pain, 5 = moderate pain, and 10 = intense pain). All adverse events were recorded throughout the study period.

**TABLE 1:** Clinician acne erythema assessment scale.

Score	Severity level	Description
0	Clear	Clear skin with no signs of erythema
1	Almost clear	Slight erythema
2	Mild	Mild erythema
3	Moderate	Moderate erythema
4	Severe	Severe, marked erythema

**TABLE 2:** Modified investigator's global assessment for acne severity.

Grade		Description
0	Clear	No inflammatory lesions and no comedones ±Residual hyperpigmentation or erythema
1	Almost clear	Few small papules and few scattered comedones
2	Mild	Easily recognizable; < 50% of the half face Some papules/pustules and some comedones
3	Moderate	> 50% of the half face Many papules/pustules and many comedones ±One nodule
4	Severe	Entire the half face Numerous papules and pustules and comedones Few nodules and cysts

#### 2.4.2 Objective Assessments

Hemoglobin (Hb) level inside the skin was captured and analyzed using Antera 3D (Miravex, Dublin, Ireland) at every visit.

### 2.5 Statistical Analysis and Graphic Presentation

Descriptive statistics were reported using appropriate formats, including counts (percentages), means ± standard deviations (SD), and medians with their ranges (minimum to maximum). To compare means across multiple groups, mixed‐effects models were used. For longitudinal analyses, a linear mixed‐effects model was applied with subject ID included as a random intercept to account for within‐subject correlation inherent to the split‐face design. Fixed effects included treatment (laser vs. AV gel), time, and the treatment × time interaction. This approach appropriately models paired repeated‐measures data. Categorical data differences were evaluated using the Chi‐squared test. The correlation analysis was performed using Spearman's correlation coefficients (*r*_s_). All statistical computations and graphical displays were carried out using GraphPad Prism version 10.2.2. A *p*‐value < 0.05 was considered statistically significant.

### 2.6 Language Model Usage

We used AI language models, specifically ChatGPT 5.2 and Gemini 3, to refine the language, improve readability, and correct grammatical errors in this manuscript. The authors are solely responsible for all scientific content, conclusions, and any remaining errors.

## 3 Results

### 3.1 Twenty‐Nine Patients with Bilateral AE Completed the Study

A total of 30 patients were included in the study. One female patient was withdrawn after 2 laser sessions due to being unable to attend scheduled sessions and follow‐up appointments. Among 29 subjects, 27 were female (93.1%) and 2 were male (6.9%). Nineteen subjects (65.5%) were Fitzpatrick skin type III, while the remaining 10 subjects (34.5%) were skin type IV. The median age was 29 years (range: 21–43 years). The median duration of AE was 6 months (range: 1–120 months).

### 3.2 The 589/1319 Nm SSDW Laser Therapy Demonstrated a Clinical Response after the First Treatment Session

**FIGURE 1:**
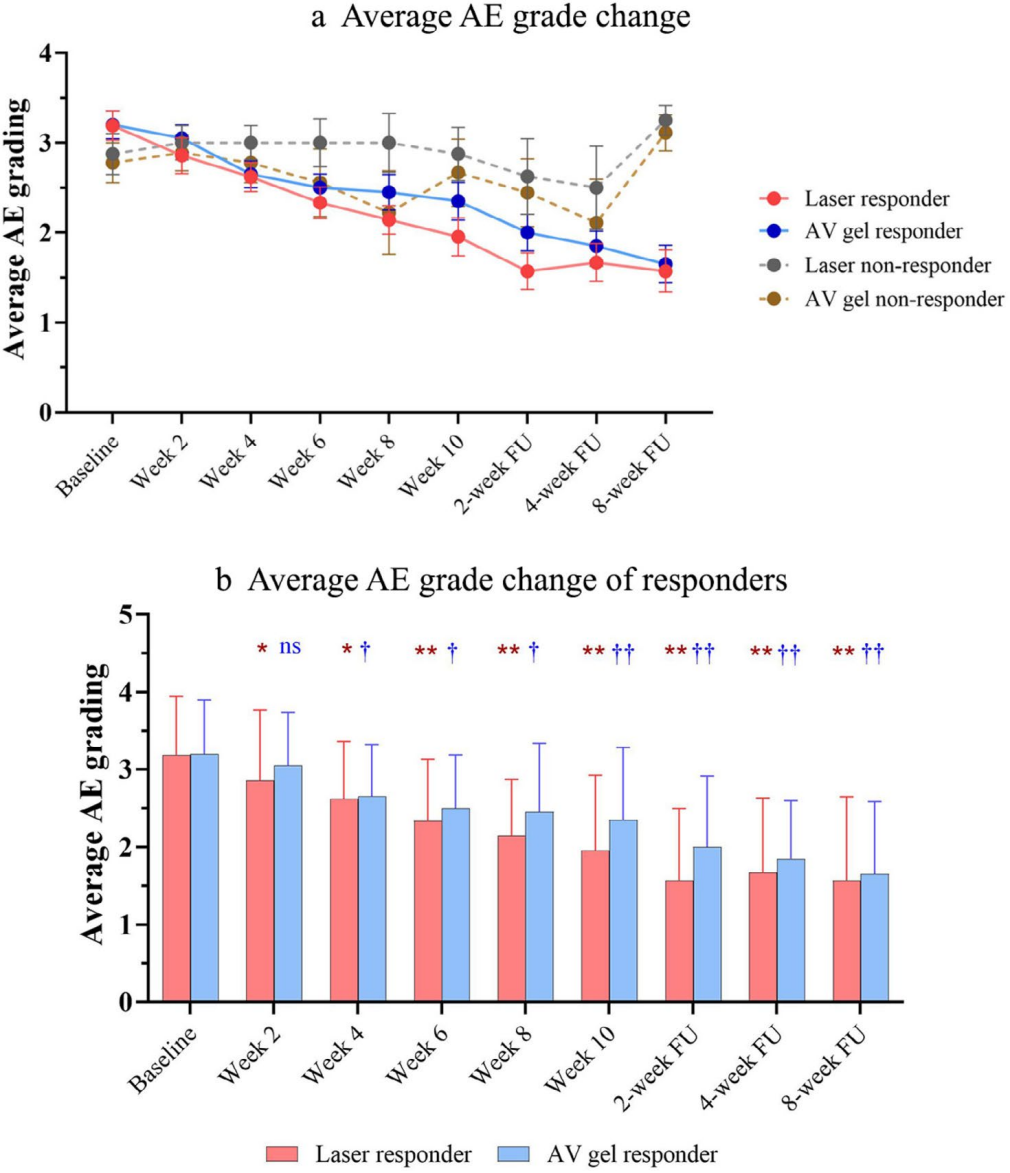
AE grade change. (a) Change of average AE grade of both responders and non‐responders. (b) Average AE grade change of responders. Statistical significance is shown for the statistical analysis, at each time point compared to baseline (*n* = 21 laser responder group; *n* = 20 for the AV responder group). * or † *p* < 0.05, ** or †† *p* < 0.0001. AE, acne erythema; AV, *Aloe vera* ; FU, follow‐up.

Response was defined as facial sides demonstrating at least a one‐grade improvement in AE grade at the 8‐week follow‐up. Laser responses were observed in 21 of 29 (72.40%) laser‐treated sides, compared with 20 of 29 (68.97%) AV gel–treated sides. Among responders, the mean AE grade decreased from 3.3 to 1.65 (50.96%) on the laser‐treated sides and from 3.2 to 1.57 (48.43%) on the AV gel–treated sides. The baseline characteristics of patients who responded to both laser and AV gel treatments did not differ significantly (Table 3). The characteristics of non‐responders are shown in Supplementary Table S1. AE grade reduction progressively improved among responders, whereas no significant improvement was observed among non‐responders (Figure 1a). Although no significant difference was detected between the 2 treatment groups (*p* > 0.5), a statistically significant reduction from baseline was observed earlier in the laser‐treated sides, beginning at week 2 (*p* = 0.03) after the first laser session. In contrast, the AV gel–treated sides demonstrated a significant reduction from baseline starting at week 4 after the initial application (*p* = 0.04) (Figure 1b). Among laser responders, the AE grade reductions remained statistically significant up to 8 weeks after the last laser session (Figure 1b).

**TABLE 3:** Characteristics of responders.

Characteristics	589/1319 nm SSDW laser responder	AV gel responder	*p*
Number *n* (%)	21 (72.40)	20 (68.97)	
Age (year) means (SD)	30.48 (5.96)	29.40 (4.50)	0.50^α^
Female *n* (%)	2 (9.52%)	2 (10%)	> 0.99^β^
Male *n* (%)	19 (90.48%)	18 (90%)	
Average Fitzpatrick skin type (FST) means (SD)	3.38 (0.50)	3.25 (0.44)	0.40^α^
FST III (*n*)	13 (61.90%)	15 (75%)	0.51^β^
FST IV (*n*)	8 (38.10%)	5 (25%)	
Facial oiliness (*n*)	11 (52.38%)	10 (50%)	> 0.99^β^
No facial oiliness (*n*)	10 (47.62%)	10 (50%)	
Acne erythema (AE) duration (months) median (range)	5 (1–120)	6.5 (1–120)	0.60^γ^
AE duration (months) means (SD)	19.43 (35.02)	21.45 (35.41)	0.90^α^
AE grade at baseline means (SD)	3.20 (0.75)	3.20 (0.70)	> 0.99^α^
AE grade at 8‐week follow‐up means (SD)	1.57 (1.08)	1.65 (0.93)	0.80^α^
Acne duration (months) median (range)	5 (0.5–20)	3.25 (0.5–20)	0.80^γ^
Acne duration (months) means (SD)	6.21 (5.17)	6.68 (5.22)	0.80^α^
Acne severity grade at baseline means (SD)	2.24 (0.77)	2.35 (0.75)	0.64^α^
Acne severity grade at 8‐week follow‐up means (SD)	1.62 (1.14)	1.35 (1.04)	0.70^α^

Abbreviation: SD, standard deviations.

^α^ *T*‐test.

^β^ Fisher's exact test.

^γ^ Mann–Whitney U test.

### 3.3 AE Improvement Correlated With Improved Acne Severity Among Laser Responders

**FIGURE 2:**
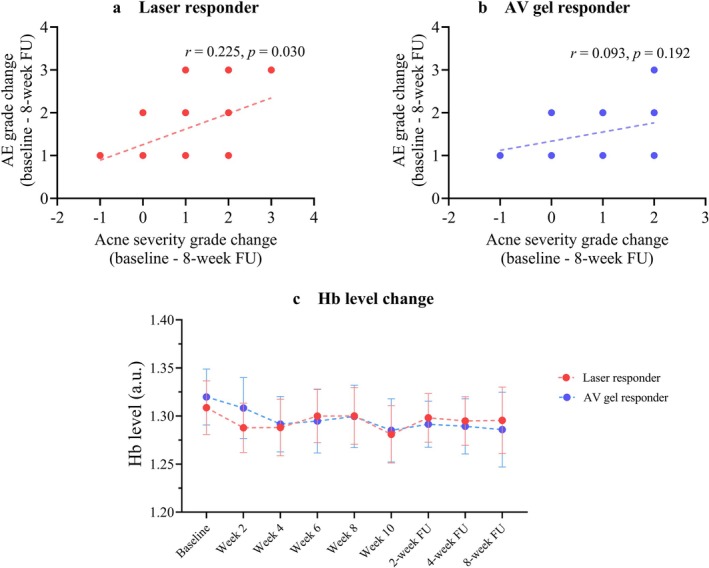
Correlation of AE change with acne severity change and Hb level at each time point. (a) Correlation of AE grade change with acne severity grade change in the laser responders. (b) Correlation of AE grade change with acne severity grade change in the AV responders. (c) Hb level change of responders. *r*_s_; spearman rank correlation coefficient. AE, acne erythema; AV, *Aloe vera* ; FU, follow‐up; Hb, hemoglobin.

The correlation between changes in AE grade and acne severity from baseline to the 8‐week follow‐up was analyzed. Results established a significant positive correlation between AE grade reduction and improvement of acne severity in laser responders (*r*_s_ = 0.47, *p* = 0.03) (Figure 2a). Although AV gel responders also showed this trend, it did not reach statistical significance (*r*_s_ = 0.30, *p* = 0.19) (Figure 2b). However, when comparing the change in AE grade between laser responders and AV gel responders, no statistically significant differences were observed at any follow‐up visit (*p* > 0.05).

Objective measurement of the Hb level within the skin, captured by the Antera 3D camera, was analyzed. Although the Hb level showed a slight decrease over time, this reduction did not achieve statistical significance, and no significant difference was observed between both treatment groups (Figure 2c).

### 3.4 589/1319 Nm SSDW Laser Treatment Satisfaction and Adverse Events

**FIGURE 3:**
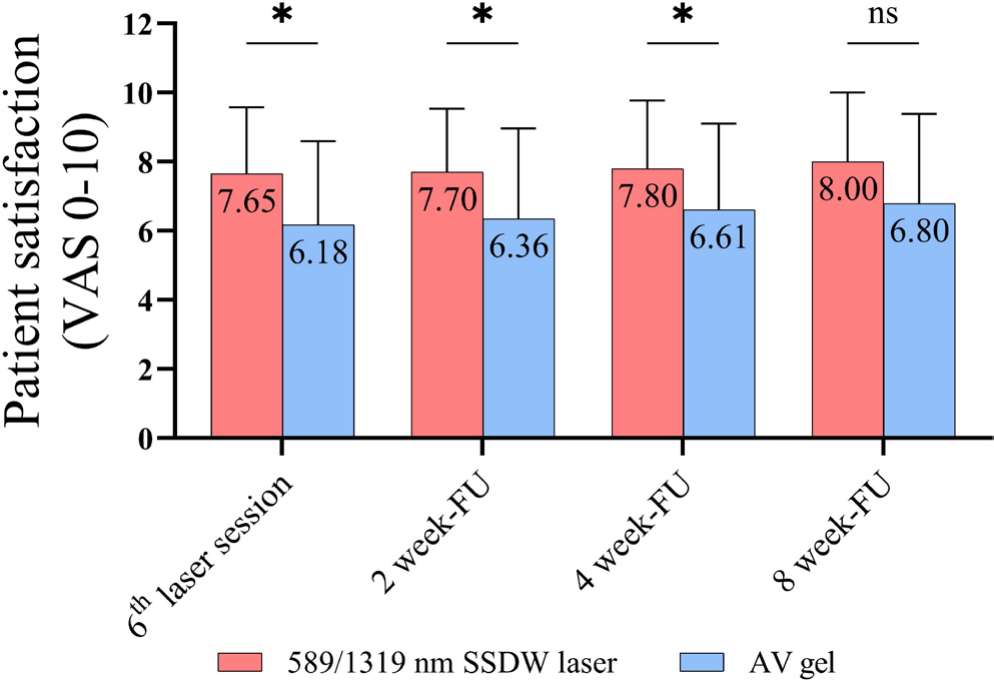
Patient satisfaction. The statistical analysis was compared between groups. *n* = 29 each group. * *p* < 0.05. AV, *Aloe vera* ; VAS, visual analog scale.

The pain VAS score for the laser treatment was 1.52 out of 10. Patients reported transient mild erythema subsiding within an hour and a warm sensation, which resolved within 30 min. Both of which were the intended endpoints of the laser treatment. Two of the 29 patients (6.90%) developed severe acne vulgaris (extensive inflammatory papules and pustules without nodules) on both facial sides during week 14 (2 weeks after the final laser treatment). Both cases were treated with topical acne therapy (5% benzoyl peroxide and clindamycin solution) and continued to complete the study. Their acne improved after the 8‐week follow‐up. These 2 participants were classified as non‐responders in both the *Aloe vera* and laser groups (Figure 1b), and their data were retained in the analysis. No other adverse events were reported for either the laser or AV group. Patients rated a significantly higher satisfaction score with laser treatment than with topical AV gel at all time points, which persisted with statistical significance up to 4 weeks after completing the 6 laser sessions (Figure 3).

## 4 Discussion

The underlying mechanisms of AE are not entirely understood, but it is thought to result from the dilation of microvascular structures in the dermis and the thinning of the epidermis during the healing process, which enhances the prominence of redness [3]. Although some AE lesions may gradually improve over time, some may persist and can result in PIH and scarring. Our study assessed the efficacy of the 589/1319 nm SSDW laser for AE treatment by comparing it to skin‐soothing AV gel. The results showed that 72% of laser treated sides achieved at least a 1‐grade AE improvement compared with 69% of AV gel sides. However, the laser demonstrated an earlier clinical response with minimal treatment discomfort, no downtime, and was associated with a reduction in acne severity and higher patient satisfaction. We analyzed the characteristics of responders to optimize patient selection for either 589/1319 nm SSDW laser or AV gel in AE treatment. The results showed no significant difference in patient characteristics between laser responders and AV gel responders; both typically presented with moderate erythema and concurrent mild acne. Interestingly, oily and non‐oily skin types were equally represented among both laser and AV gel responders. Discussion of the effectiveness of the 589/1319 nm SSDW laser and AV gel in the treatment of AE are as follows.

**FIGURE 4:**
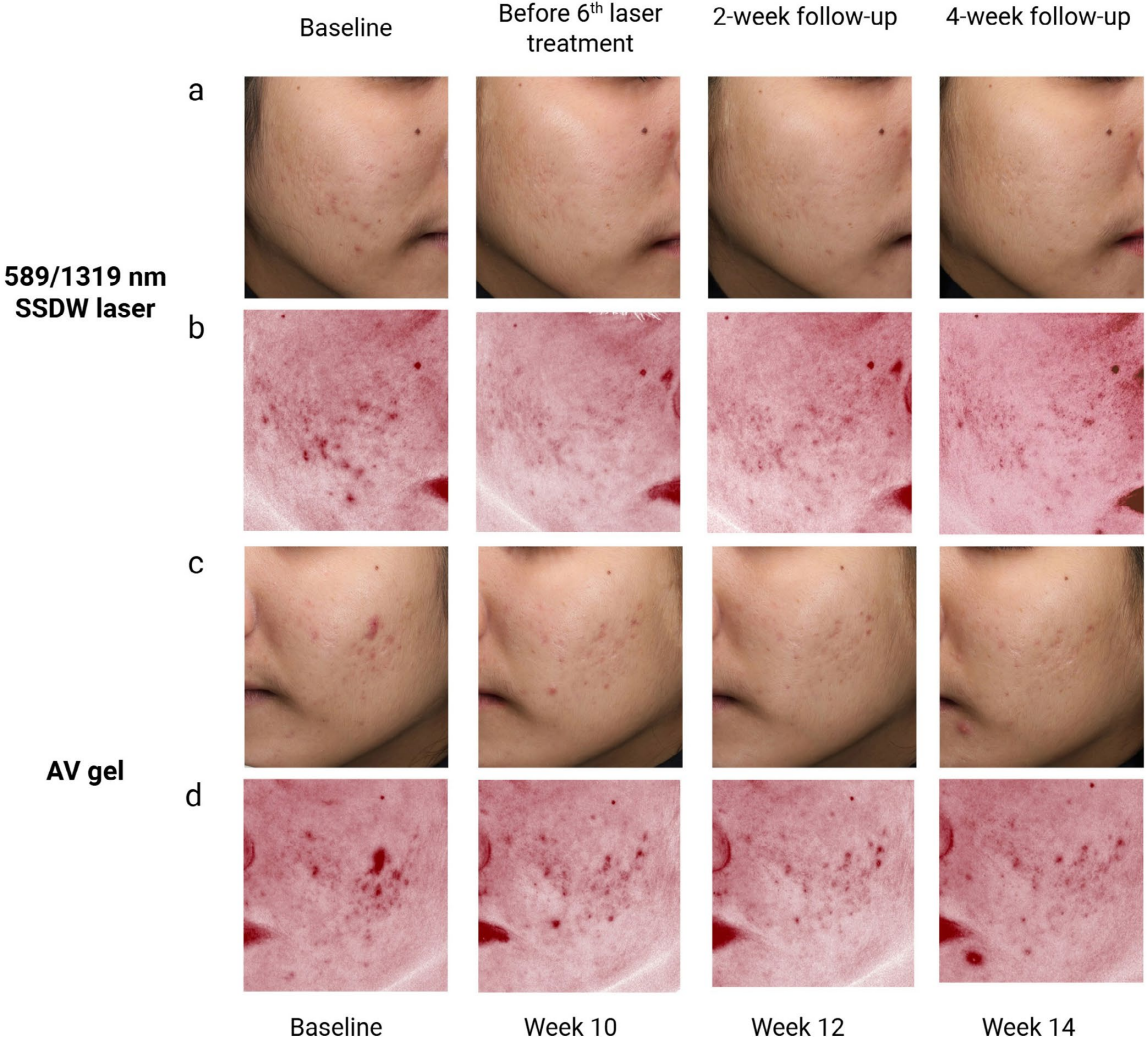
Clinical and Hb level photographs of responders. Clinical and Hb‐captured photos from a responder are presented. The laser‐treated side is shown in panels (a–b), while the AV gel‐treated side is shown in panels (c–d). The 589/1319 nm SSDW laser treatment was administered at 2‐week intervals for a total of 6 sessions. The AV gel was applied twice daily over an 18‐week study period. AV, *Aloe vera* ; FU, follow‐up; Hb, hemoglobin; SSDW, solid‐state dual wavelength.

The 589 nm wavelength, with a penetration depth of up to 1.25 mm, can effectively target superficial microvasculature by selectively absorbing oxyhemoglobin, resulting in photocoagulation of dilated blood vessels with subsequent reduction in redness. While the 1319 nm wavelength penetrates 2 mm into the dermis and selectively targets water, which promotes collagen remodeling, strengthens the dermis and thus may diminish the visibility of erythema [20]. In addition, the generated heat can reduce sebaceous gland activity [18]. Our results demonstrated that among laser responders, laser treatment achieved significant reductions in AE grade 2 weeks earlier than the AV gel (without significant difference at the primary endpoint). This also correlated with a reduction in acne severity grades. The effects were shown to persist for at least 8 weeks after completing laser sessions. Photographs of laser and AV gel responders are shown in Figure 4.

Our study findings align with previous research. Cohen DK et al. [16] evaluated the efficacy and safety of a 589 nm solid‐state laser for treatment of facial erythema in 24 participants. Their study reported a statistically significant 31% reduction in facial erythema on both sides of the face, as assessed by investigators, with minimal side effects of temporary erythema after treatment. Furthermore, a study by Lim J [21]. investigated the combined use of the 589/1319 nm SSDW laser and topical retinoids (0.1% adapalene or 0.025% tretinoin) in 40 Asian patients with moderate to severe acne vulgaris. Patients were assessed at baseline, monthly over 5 treatment sessions, and 1 month after the final laser session. All patients reported acne improvements, with 95% having improvements described as much or very much. Among the 29 patients with erythema, the investigator evaluated that 65.5% experienced over 75% improvement of erythema. Although this study proposed the effectiveness of this combined approach for managing acne and AE, it lacked a control group. In another small study, four fractional 589/1319 nm SSDW laser sessions in 9 patients with moderate to severe acne vulgaris decreased inflammatory acne lesions and also improved skin redness in 66.7% (6/9) of participants at the end of treatments [22]. These studies demonstrate the effectiveness of this dual‐wavelength laser, targeting both the vascular and dermal components, in improving AE. Moreover, results of our study confirmed that the acne erythema responding to laser treatment additionally showed improvement in acne severity grade. This confirms our previously published study that verified the use of 589/1319 SSDW laser as an effective adjunctive treatment for inflammatory acne [18]. Although different laser parameters and inclusion criteria were used in the present study, we observed an association between changes in AE grade and changes in acne severity grade.

Our results also found that topical AV gel is also effective in the reduction of AE. Topical AV provided improvements in erythema grading possibly due to its anti‐inflammatory, antioxidative, moisturizing, and antibacterial properties. These attributes help soothe the skin and promote epidermal barrier function [10, 11]. Hajheydari Z. et al. [9] conducted a randomized, double‐blind, 8‐week trial to evaluate the efficacy and safety of combining 0.05% tretinoin (TR) cream with 50% AV gel (TR/AV group) compared to TR and placebo vehicle (TR/placebo group) in 60 subjects with mild to moderate acne vulgaris. The combination TR/AV therapy demonstrated significantly superior efficacy with reductions in non‐inflammatory, inflammatory, and total lesion scores compared to the control group. Furthermore, erythema severity was significantly lower in the TR/AV group than in the TR/placebo group (*p* = 0.046), suggesting the additional effects of AV gel in reducing AE. In this study, the reduction of AE from AV gel was slower than the laser because it primarily provides soothing benefits through a general biological support system, whereas the 589/1319 nm SSDW laser directly targets the vascular and dermal components. Due to these different mechanisms, the combination of both 589/1319 nm SSDW laser and AV gel may possibly offer greater or faster AE improvement and/or acne severity reduction. However, further studies are needed to confirm this.

Our study confirmed the favorable safety of the 589/1319 nm SSDW laser treatments in Asians with skin type III‐IV. No serious adverse events occurred throughout the study. The dual wavelength laser treatments were minimally painful (mean pain score: 1.52/10, not requiring anesthetic cream) and downtime‐free. While purpura and pain are common side effects associated with laser treatment of vascular lesions [17], these were not found in our findings. Of note, 2 of the 29 subjects developed severe acne on both laser‐treated sides and AV gel‐treated sides of the face during the follow‐up period. It is possible that their acne was not well‐controlled. Patients with moderate acne vulgaris have a propensity to develop severe acne, and topical treatment alone might have been insufficient, necessitating more intensive acne treatment. However, both patients continued to participate in the study and completed all follow‐up visits. Their data was included in the statistical analysis as non‐responders in both the laser and AV gel groups, which may have influenced the study results.

Regarding patients' satisfaction with both AE treatments, patients reported significantly greater and sustained satisfaction of the laser treatment side than the AV gel. Furthermore, the statistically significant improvements persisted for up to 4 weeks after discontinuing laser treatment, suggesting a 4‐week interval for additional laser treatment if needed.

While this study introduces promising options for AE treatment, it is essential to acknowledge its limitations. Firstly, uncontrolled acne or the emergence of new acne lesions during the follow‐up period could have confounded the precise evaluation of AE improvement. Secondly, the lack of long‐term follow‐up. Thirdly, the small sample size and single‐ethnicity methodology limit the broader generalizability of these findings; post hoc analysis based on the observed paired difference yielded a statistical power of approximately 74% (two‐sided α = 0.05), indicating moderate but slightly suboptimal power. Finally, despite using designated treatment endpoints of mild erythema for 589 nm and a warm heat sensation for 1319 nm, the optimal laser parameters in Asians to achieve superior outcomes remain an important avenue for future research.

In conclusion, our study shows that both the 589/1319 nm SSDW laser and topical soothing AV gel are effective treatments for improving AE. While AV gel offers anti‐inflammatory, antibacterial effects and skin healing benefits, the laser provides earlier improvement, reduces acne severity without downtime, thus higher patient satisfaction. This is likely because the dual laser wavelengths directly target vascular components, sebaceous glands, and promote dermal remodeling. Therefore, the 589/1319 nm SSDW laser may serve as a useful early adjunctive treatment for acne erythema, particularly in Asian patients, including those with active acne, especially individuals seeking treatment with reduced discomfort or without downtime. Patient selection is essential to optimize outcomes, and further studies are needed to define optimal laser treatment parameters, number of laser sessions, and long‐term efficacy.

## Author Contributions

Suphagan Boonpethkaew: investigation, formal analysis, software, visualization, writing‐original draft, writing‐review and editing. Pimsiri Anansiripun: investigation, resource, formal analysis, visualization, writing‐original draft. Warittha Maitrisathit: conceptualization, methodology, investigation, resources. Sonphet Chirasuthat: investigation, formal analysis, software. Yanisa Ratanapokasatit: conceptualization, methodology, investigation, resource. Panrudee Wechsuruk: investigation, resource. Penpun Wattanakrai: conceptualization, methodology, investigation, writing‐review and editing, project administration, supervision.

## Funding

The financial support was provided by the Division of Dermatology, Department of Medicine, Faculty of Medicine, Ramathibodi Hospital, Mahidol University, Thailand.

## Ethics Statement

This study was approved by the University Institutional Review Board for Ethics in Human Research, Faculty of Medicine Ramathibodi Hospital, Mahidol University (COA.MURA2022/747). Subjects provided signed informed consent for participation and photographs.

## Conflicts of Interest

The laser device used in this study was provided on a short‐term loan from the Thailand distributor (Laser Aesthetics Co. Ltd., Bangkok, Thailand) as material support. The distributor had no influence on the study design, data collection, analysis, interpretation or manuscript preparation; all of which was conducted independently by the authors. The authors declare that they have no other conflicts of interest.

## Supporting information


**Table S1:** Characteristic of non‐responders.

## Data Availability

The data that support the findings of this study are available from the corresponding author upon reasonable request.
