# Supplementary material for: Efficacy and Safety of a Dual‐Wavelength 589/1319 nm Laser for the Treatment of Acne Erythema: A Split‐Face Randomized Controlled Trial
Source: J Cosmet Dermatol. 2026 May 5;25:e70894. doi: 10.1111/jocd.70894 (PMC13145309; doi:10.1111/jocd.70894)
Supplement: Supplementary file 1 — Table S1: Characteristic of non‐responders. [file JOCD-25-e70894-s001.docx]

**Supplementary**

**Table S1** Characteristic of non-responders

| **Characteristics** | **589/1319 nm SSDW laser non-responder** | **AV gel non-responder** |
| --- | --- | --- |
| Number n (%) | 8 (27.59%) | 9 (31.03%) |
| Age (year) means (SD) | 26.88 (4.16) | 26.67 (7.38) |
| Female n (%) | 8 (100%) | 9 (100%) |
| Male n (%) | 0 | 0 |
| Average Fitzpatrick skin type (FST) means (SD) | 3.25 (0.46) | 3.56 (0.44) |
| FST III (n) | 6 (75.00%) | 4 (44.44%) |
| FST IV (n) | 2 (25.00%) | 5 (55.56%) |
| Facial oiliness (n) | 7 (87.50%) | 8 (90.67%) |
| Non oiliness (n) | 1 (12.5%) | 1 (11.11%) |
| Acne erythema (AE) duration (month) median (range) | 8 (1−36) | 6 (1−36) |
| AE duration (month) means (SD) | 13.63 (12.76) | 9.78 (12.22) |
| AE grade at baseline means (SD) | 2.88 (0.64) | 2.78 (6.67) |
| AE grade at 8-week follow-up means (SD) | 3.25 (0.46) | 3.11 (0.60) |
| Acne duration (month) median (range) | 5.5 (3−10) | 3.56 (1−10) |
| Acne duration (month) means (SD) | 6.5 (3.07) | 5.44 (3.06) |
| Acne severity grade at baseline means (SD) | 2.13 (0.83) | 3 (0.87) |
| Acne severity grade at 8-week follow-up means (SD) | 2.63 (0.92) | 2.44 (1.01) |
